# Analysis of the kinematic pattern of free throw by wheelchair basketball athletes: a systematic review

**DOI:** 10.1371/journal.pone.0317495

**Published:** 2025-02-06

**Authors:** Hugo Vinícius de Oliveira Silva, Karina Santos Guedes de Sá, José Irineu Gorla, Anselmo Athayde Costa e Silva, Dernival Bertoncello

**Affiliations:** 1 Federal University of Triângulo Mineiro, Universidade Federal do Triângulo Mineiro,; 2 Brazilian Paralympics Academy, Academia Paralímpica Brasileira,; 3 College of Physical Education, State University of Campinas, Faculdade de Educação Física, Universidade Estadual de Campinas,; 4 Laboratory of Human Movement Analysis, Federal University of Triângulo Mineiro,Laboratório de Análise do Movimento Humano, Universidade Federal do Triângulo Mineiro,; 5 Laboratory of Adapted Physical Activity, Federal University of Pará,Laboratório de Atividade Física Adaptada, Universidade Federal do Pará; Opole University of Technology: Politechnika Opolska, POLAND

## Abstract

Athletes are stratified into classes that range from 1.0 to 4.5 points subdivided at intervals of 0.5 points in the Wheelchair Basketball (WCB) modality. The discussion regarding the non-utilization of quantitative data based on functional classification (FC) in WCB has become considerably important for research due to its impact on athletes’ careers. The aim of this systematic review was to verify the kinematic patterns of the free throw performed by WCB athletes of distinct FC. After researching four online databases, only 7 out of 68 studies fulfilled the inclusion criteria. High-class players (classes 3.0 – 4.5) presented a throw pattern, with a greater angle on the shoulder joint, on the angular speed of the wrist, and the height of the ball throw; low-class athletes (classes 1.0 – 2.5) exhibited a pushing pattern, with greater speed and throw angles, greater angular speed at the shoulder and elbow joints. Thus, inter-individual differences allow for the stratification of players into distinct classes and serve as relevant tools for increasing accuracy and performance despite different disabilities. Further studies are necessary to explore the associated patterns for each FC.

## Introduction

The functional classification system of Wheelchair Basketball (WCB) considered by the International Wheelchair Basketball Federation (IWBF) aims to group athletes into sports classifications. In this modality, athletes are stratified in classes that range from 1,0 to 4,5 points subdivided at each 0,5 points. This guarantees that impairment is not a determining factor on the sports performance and result [1,2].

The classification is conducted mainly from non-quantitative tests related to the volume of action and are directly related to the control of trunk/volume action and the influence of lower limb and upper limb limitations on sports fundaments [1,2]. This can cause different interpretations among classifiers, making the method subjective, which may directly impact the athletes’ yield and career.

Then, since the Tokyo 2020 Paralympics Games, the IWBF classification manual has been going through adjustments according to the IPC Code of Classification [3]. It defines that the evaluation stages of the functional classification must be developed from multidisciplinary scientific research, based on evidence in relation to the impairment with performance. Thus, the classification must involve the impairment in the performance of the game [4].

Currently, such classification is conducted under the approval of the MIC (Minimum Impairment Criteria) and not directly to define the players’ functional classes [1]. For that to happen in WCB, the IWBF functional classification system must minimize its classification according to the general function [2] and seek other assessment forms that present objective data over the players. Thus denoting the influence of such data on sports performance, allowing for the distinction of players into functional classes [4].

Therefore, the literature brings some variables as possible descriptors of performance and functional class. However, the biomechanics analysis has been considered the ideal scientific evidences for the validation of different methods of evidence-based classification [5], when they directly relate to sports performance [6]. Thus, it is necessary to analyze the variables which demonstrate to be important to the success in the match, as throws, assistances and fouls received [7].

Various strategies are employed by athletes to minimize the impact of disability on the execution of offensive and defensive principles in wheelchair basketball (WCB). In this context, different shooting position patterns are viewed as a way to increase the success rate of athletes from various functional classes [8,9]. Similarly, inter-individual differences in free throw shooting technique between successful and unsuccessful attempts among players from different classes [10].

Therefore, the higher shooting percentages, which includes the free throw, offer an advantage for the team to have victory in the match [7,11,12]. Furthermore the performance on free throw exhibits variability in scoring percentage according to the functional classes and players’ gender [7,13,14]. For it is a closed skill, that is conducted in a stable and predictable environment, free-throw shots favor, due to less intra-individual variability of movement, its biomechanics analysis.

The joint kinematic and ball-throw parameters become important to denote intra-individual and inter-individual evidence which allow the characterization of functional classes as it is discussed the importance of the throw in a game. It makes the current method more robust and credible [6], apart from providing additional information to players and coaches who seek a better understanding and development of the sports performance.

It is also important to consider body variations that influence biomechanical parameters during throwing for better effectiveness of the method. Aspects such as shoulder pain are common in this population due to the increased load and repetitive stress caused by both sports activities and the daily use of the wheelchair or orthopedic accessories [15,16]. In this way, sports performance is directly affected and may worsen without proper monitoring of internal and external loads [17,18]. Thus, aspects such as trunk and upper limb strength, the relationship with internal loads, and the procedures performed during warm-up periods must be taken into consideration when analyzing factors directly related to performance and accuracy, as in the case of shooting [19].

The applications of the motor gesture performed in the free throw to obtain performance may require adjustments for WCB players and the recommendations for throw based on Olympic basketball general principles may become expendable for this specific population [20]. Furthermore, in order to remedy incompatibilities between the IWBF Manual of Classification and the IPC Code of Classification of Athletes, information to determine the eligibility of athletes and evidence to group up athletes with a similar general function into classes is necessary [2]. Therefore, this study aimed to review the existing literature to characterize the kinematic pattern of free-throw shots performed by wheelchair basketball athletes of different functional classes and discuss which features are relevant for the performance of free throw for this population.

## Methods

### Search strategy

The main question was developed through the PICO strategy [21]: Participants: wheelchair athletes; Interest: Research on throw in wheelchair basketball; Comparison: functional classes; and Results: throw pattern is different among athletes of different classes.

### Selection criteria

Were considered eligibility criteria for this paper: cross-sectional, observational and longitudinal studies of texts in English and studies which have applied tests to evaluate the free throw and made comparisons between among different functional classes. This research does not include studies which were characterized as review articles, nor evaluated the free throw or not divided the athletes into classes.

### Procedure

**Fig 1:**
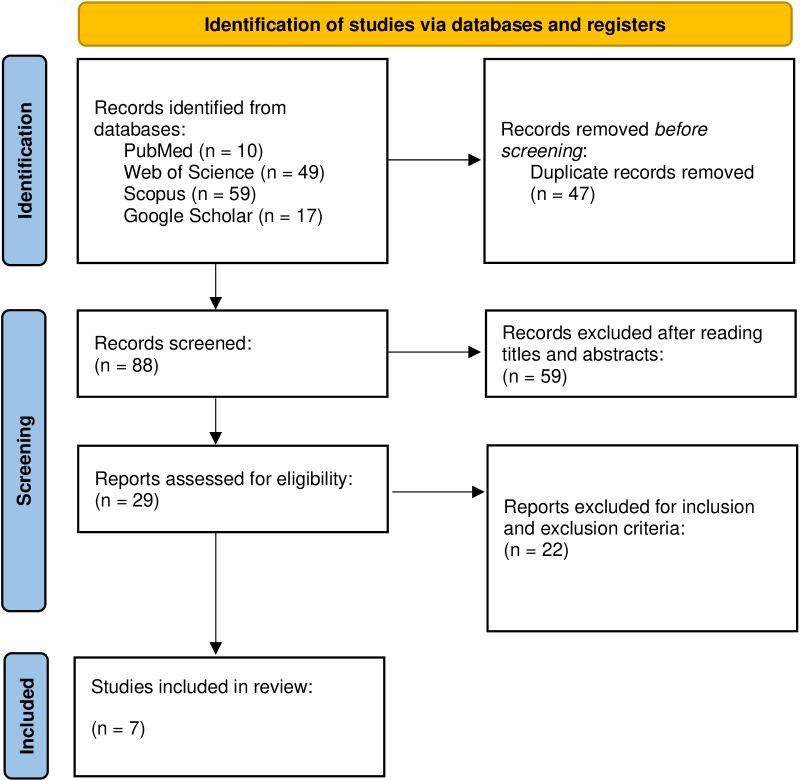
Prisma flow diagram. *From:* page MJ, McKenzie JE, Bossuyt PM, Boutron I, Hoffmann TC, Mulrow CD, et al. The PRISMA 2020 statement: an updated guideline for reporting systematic reviews. BMJ 2021;372:n71. https://doi.org/10.1136/bmj.n71.

There were conducted searches in English language on the electronic databases PubMed/ Medline, Web of Science, Scopus and Google Scholar, between December, 2021 and January, 2022, with an update in January, 2025. using the following search strategy: “wheelchair basketball” AND “throw”. After searching, two researchers properly trained selected the articles independently, excluding duplicated. Decision making was based on the titles and abstracts of the articles and inclusion criteria previously described (Fig 1). A third researcher solved occasional divergences.

**Fig 2:**
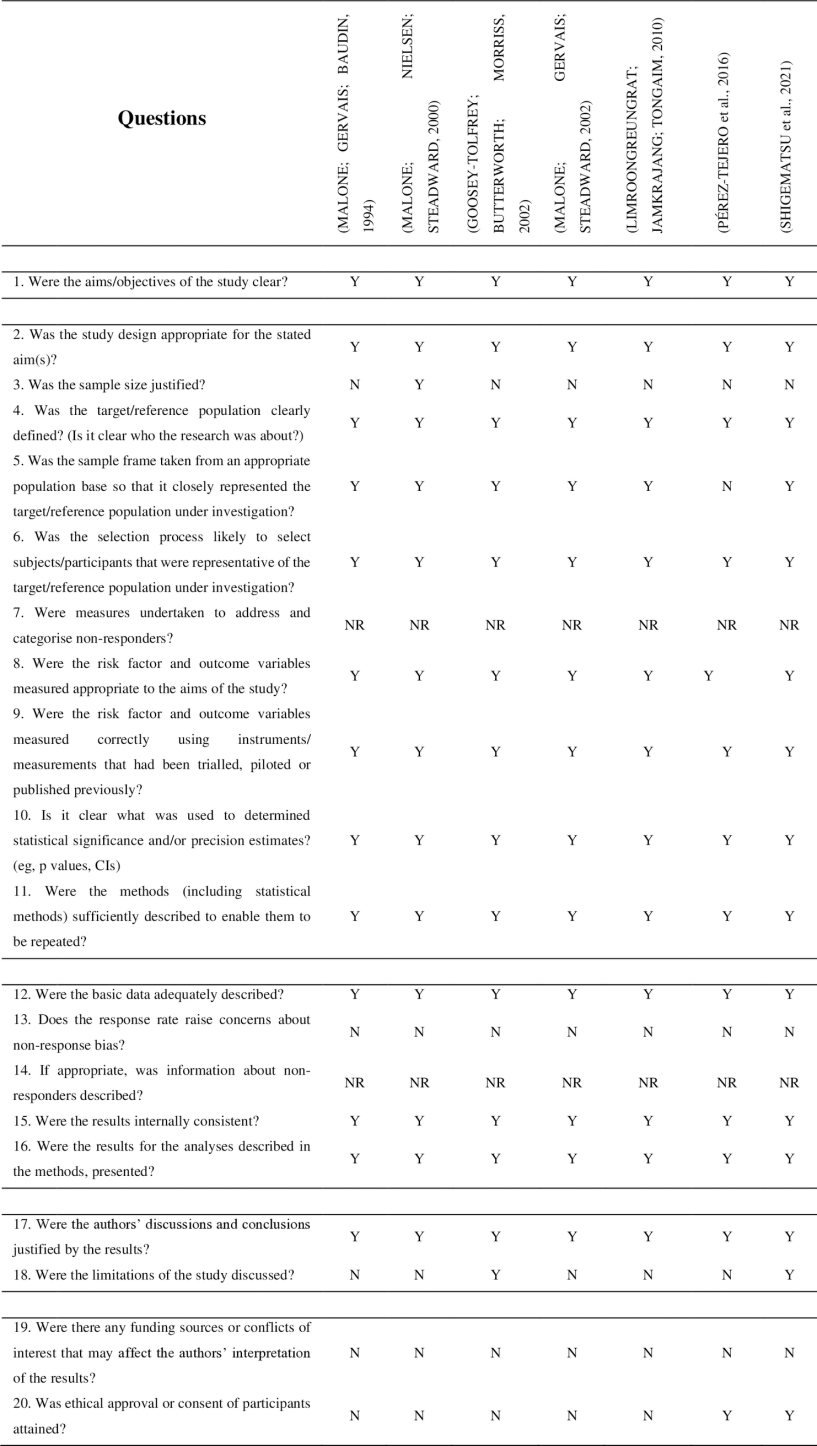
Appraisal for cross-sectional studies (AXIS tool). Legend: **Y** =  Yes, **N** =  No and NR =  Not Reported.

The methodological quality of the studies was assessed by the tool Appraisal for Cross-Sectional Studies (AXIS tool) [22]. This tool is composed of twenty questions which evaluate the bias risk of cross-sectional studies as described in Fig 2.

The research in the literature resulted initially in 135 articles. After the removal of 47 duplicated found on the databases or with different research terms, were examined 88 remaining articles to verify which fulfilled the inclusion and non-inclusion criteria. After reading the title and abstract, were removed 59 articles, leaving 29 articles for full-text evaluation. The criteria application during the review resulted in the non-inclusion of 22 articles. Another article was excluded during the data extraction; the remaining 7 articles from which the data were extracted were synthesized in the results.

### Analysis

Thereafter, the data extraction was initialized, conducted independently by two researchers and, when there was any inconsistence, a third researcher sorted them out. Through data extraction, were conducted a descriptive statistic of the results by means and standard deviations.

## Results

All seven studies selected are quantitative and cross-sectional [13,23–28]. All studies used video analysis to characterize the throws from distinguished forms of data collection. Some studies collected data on free throws that occurred during an official competition [13,23,25] and gathered data during a training session. The former involved five series of two intercalated free-throw attempts with half-court runs, with two clear throws chosen randomly for analysis, while the latter involved the collection and analysis of two successful free throws [24,27]. Other studies collected attempts and analyzed two throws considered clean [26] and conducted the collection of twenty free throws [28]. Three of these studies conducted complementary assessments, evaluating the ball-into-the-basket standard from a schematic diagram developed specifically for this study [13,25,28].

The total number of individuals amongst the studies was 216 athletes, from which 92.59% were identified as players representing their national teams, all of the male gender. Of which, twenty are class 1.0, thirty-two are class 2.0, twenty-two class 3.0, thirty-six class 4.0 and 116 did not have a defined quantity of individuals for each class. Five works evaluated class 1.0 players [13,23,25,27,28]. While four studies evaluated class 2.0 players [13,24,25,28],

4 works evaluated class 3.0 players [13,25,26,28] and six other works evaluated class 4.0 players [13,24–28]. The summary of all studies is presented on Table 1.

**Table 1:** Summary of study characteristics for included studies.

Study	Aim	Sample	Country	Age (yrs)	Cohort	Sport level	Data Collection	Key results
MALONE; GERVAIS; BAUDIN, 1994	To develop a better understanding of FT (free throw) performed by wheelchair basketball class 1.0 players and describe the kinematic differences on successful and unsuccessful FT attempts.	n = 7 male (all class 1.0)	Canada	Not informed	National Team Players	Professional	2 Panasonic SVHS cameras	Taking into account both shoulder and elbow angle speeds by each athlete, the data suggest two distinctive patterns. The majority of the players seemed to use a pushing movement for the FT, while others used a sequential or throwing pattern. Head movement ranged from 0 to 9 degrees before the throw, with a tendency to less movement at successful shots.
MALONE; NIELSEN; STEADWARD, 2000	To provide a technique to describe the result of FT (free throw) in addition of the traditional dichotomy and employ it determining the characteristics of FT shot at a wheelchair basketball competition.	n = 116 male (all classes, n does not define n of each class)	Canada	Not informed	National Team Players	Professional	Visual observation and recording in a schemal diagram	Class 4 players hit over half (56%) of all free throw attempts during the tournament. 43% (n = 317) of all attempts observed (successful and unsuccessful) were initially short, and the majority (70%) resulted in missed shots. Chi-square test results indicated that there was no significant association between the player’s class and shot pattern
GOOSEY-TOLFREY; BUTTERWORTH; MORRISS, 2002	To determine the characteristics of FT shot at a male wheelchair basketball competition.	n = 15 male (4 class 2.0; 6 class 2.5; 3 class 4.0; 2 class 4.5)	Great Britain	18 - 39	National Team Players	Professional	2 Panasonic videocameras (Model AG-DP800He; 50 Hz)	Ball-throw angle was 58 for both groups. Group 2 (higher-class athletes) threw the ball from a significant higher point than those of Group 1 (lower-class athletes) (1,57–0,12m v 1,78–0,17m; p < 0,05). Although not significant, there were found the following tendencies: Group 1 denoted greater ball release speed and wrist angle speed at releasing. Group 2 generated greater flexion angle speed on the shoulder at releasing.
MALONE; GERVAIS; STEADWARD, 2002	To identify the differences in the ball release parameters (height, angle and speed) among the classes to determine the necessary technique.	n = 67 male (7 class 1.0; 16 class 2; 18 Class 3; 26 Class 4)	Canada	Not informed	National Team Players	Professional	2 Panasonic SVHS (Super Video Home System)	Significant differences were observed among the players’ classes on the FT shot mechanics. Class 1 and 2 players tended to throw the ball from a lower height, with higher throw angle and speed. They presented a lesser angle flexion on the shoulder and a greater maximum speed on the shoulder and elbow. Class 1 and 2 clear shots demanded greater precision in relation to throw and angle speed, but the resulting ball trajectory denoted a greater error margin than the observed shots by superior classes. However, based on the general shot percentage, superior classes do not seem to take advantage of the benefits previewed provided by a higher ball-throw height.
LIMROONGREUNGRAT; JAMKRAJANG; TONGAIM, 2010	To compare successful kinematic free-throw shot differences in wheelchair basketball among high and low-class Thai professional athletes.	n = 05 omens (1 class 3.0; 1 class 3.5; 3 class 4.5	Thailand	18 - 35	National Team Players	Professional	3 high-speed digital videocameras (Casio Exilim Pro EX-F1, Japan).	High-class players had a greater shoulder and elbow movement amplitude than the low-class group, while the low-class group used greater movement amplitude than the high-class group. As the low-class group used a lesser movement amplitude on the shoulder and elbow joint, they were able to compensate the wrist joint. However, there were not found significant statistic differences of the angles of the upper limb between the two groups (p < 0,05).
PÉREZ-TEJERO et al., 2016	To analyze the free throw of two WCB players class 1.0 and 4.5 through the biomechanics techniques, defining specific variables in relation to efficiency and influence IOF different body segment, in order to suggest training proposals.	n = 2 male (1 class 1.0 and 1 class 4.5)	Espanha	28 - 36	2 WCB Professional players	Professional	Three-dimensional photogrammetryvideo câmera (Panasonic DP – 800 H)	Ball-throw height and gravity-center height at the ball throw were higher by class 4.5 (1.578 m and 0,689 m, respectively) than by class 1 player (1.278 m and 0,504 m). The use of distinctive segments throughout the shot was different, outstanding a more pushing pattern (class 1) in contrast to a more sequential pattern (class 4.5), denoting similar temporal parameters (assembly and releasing time) by both players.
SHIGEMATSU et al., 2021	To analyze the free throw of two WCB players class 1.0 and 4.5 through the biomechanics techniques, defining specific variables in relation to efficiency and influence IOF different body segments, to suggest training proposals. To investigate the relationship between free-throw accuracy and performance variables	n = 14 male (2 class 1.0; 3 class 1.5; 4 class 2.0; 2 class 2.5; 1 class 3.0; 1 class 3.5 and 1 class 4.0)	Japan	18 – 36	National Team Players	Professional	Two high-speed video cameras (EX-F1, CASIO COMPUTER Co., Ltd., Tokyo, Japan) - 300 Hz	A significant negative correlation was observed between the free-throw accuracy and mean pre-shot time, suggesting that participants with a shorter pre-shot time showed a higher free-throw accuracy. In addition, a significant negative correlation was found between the free-throw accuracy and variability of angular velocity of the hip at the time of ball release, indicating that the consistency of hip movement is an important factor in free-throw accuracy. There was no correlation with functional class

Six of the studies used video analysis to characterize the mechanics of free-throw shots performed by WCB athletes. Two Panasonic SVHS AG-450 cameras operating at 60 Hz, one were positioned parallel to the free-throw line and on the baseline to obtain both a side and a frontal view of the player [23,25]. Two Panasonic AG-DP800He cameras, 50 Hz were placed in front of the player and behind the baseline, on the right and the other on the left, forming an optical axis of approximately 90 degrees [24]. Three high-speed digital video cameras Casio exilim Pro EX-F1, 30 Hz, did not have their positions described [26]. High-speed analogical video cameras Panasonic DP – 800H, were positioned at a right angle in relation to their optical axis [27]. And finally, Shigematsu et al. (2021) used two Casio Computer Co EX – F1 high-speed cameras, 300Hz, one positioned at approximately four meters perpendicular to the right of the shot spot of the right-handed player and the second positioned nine meters perpendicular to the right of the ball’s movement plane [28]. Exceptionally, Malone et al. (2000) conducted a visual observation of the free throws and recorded their observations on a schematic diagram developed specially for the study to describe the ball-into-the-basket pattern.

Three studies conducted analyses of the pattern of ball throw, including height, angle and speed of the throw [24,25,27], as well as the error margin and entrance angles which were evaluated only by Malone and collaborators (2002). In relation to the joint parameters, it was measured the shoulder flexion, elbow extension and initial angle of the elbow [24–26]. Values are described in Table 2.

**Table 2:** Summary of throw parameters of the studies.

Study	Ball release height (cm)	Ball release angle (º)	Ball release speed (cm/s)	Error margin (cm)	Entrance angles (º)	Shoulder flexion (º)	Elbow extension (º)	Initial angle of the elbow (º)
**Malone et al. (1994)**	156 to 188 (Class 1);	54 to 61 (Class 1)						
**Malone et al. (2002)**	162 ± 04 (Class 1)	59 ± 2 (Class 1)	743 ± 22 (Class 1)	3,5 ± 0,9 (Class 1)	44 ± 3 (Class 1)	116 ± 8 (Class 1)	139 ± 8 (Class 1)	48 ± 4 (Class 1)
	160 ± 06 (Class 2)	58 ± 2 (Class 2)	719 ± 32 (Class 2)	2,9 ± 1,1 (Class 2)	42 ± 4 (Class 2)	123 ± 7 (Class 2)	142 ± 7 (Class 2)	46 ± 5 (Class 2)
	179 ± 13 (Class 3)	55 ± 3 (Class 3)	707 ± 30 (Class 3)	2,5 ± 1,1 (Class 3)	40 ± 4 (Class 3)	133 ± 9 (Class 3)	145 ± 8 (Class 3)	52 ± 1 (Class 3)
	184 ± 17 (Class 4)	55 ± 3 (Class 4)	699 ± 21 (Class 4)	2,5 ± 1,4 (Class 4)	40 ± 5 (Class 4)	132 ± 8 (Class 4)	143 ± 7 (Class 4)	51 ± 6 (Class 4)
**Goosey – Tolfrey et al. (2002)**	157 ± 12 (Class 2)	58 ± 2 (Class 2)	760 ± 40 (Class 2)			104 ± 36 (Class 2)	138 ± 11 (Class 2)	51 ± 5 (Class 2)
	178 ± 17 (Class 4)	58 ± 4 (Class 4)	720 ± 20 (Class 4)			123 ± 13 (Class 4)	141 ± 10 (Class 4)	53 ± 2 (Class 4)
**Limroongreungrat et al. (2010)**						128.2 (Class 3)	135 (Class 3)	
						130,5 (Class 4)	140 (Class 4)	
**Pérez – Tejero et al. (2016)**	127,8 (Class 1)							
	157,8 (Class 4)							

Yet in relation to the joint kinematic patterns, Malone and collaborators (2002) analyzed the shoulder, elbow, and wrist angle speeds, while Goosey – Tolfrey and collaborators (2002) analyzed the shoulder and wrist mean angle speed, as shown in Table 3.

**Table 3:** Summary of the angular velocity of the studies.

Study	Shoulder maximum angular speed (°/s)	Maximum elbow angular speed (°/s)	Maximum angular speed of wrist (°/s)	Mean angular speed of shoulder (°/s)	Mean angular speed of wrist (°/s)
**Malone et al., 2002**	462 ± 61 (Class 1)	957 ± 111 (Class 1)	791 ± 231 (Class 1)		
	533 ± 75 (Class 2)	888 ± 113 (Class 2)	940 ± 212 (Class 2)		
	441 ± 128 (Class 3)	798 ± 117 (Class 3)	1003 ± 175 (Class 3)		
	412 ± 89 (Class 4)	776 ± 79 (Class 4)	1038 ± 248 (Class 4)		
**Goosey – Tolfrey et al., 2002**				280,74 ± 126,05 (Class 2)	1254,77 ± 572,95 (Class 2)
				372,42 ± 74,48 (Class 4)	847,97 ± 635,98 (Class 4)

## Discussion

This systemic review analyzed quantitatively the variables which influence the performance of the free throw of each functional class of wheelchair basketball athletes. Seven studies included were heterogeneous as to the collected analyses and variables, although all of them have conducted analyses of free throws. Kinematic analysis of free-throw shots seems to be a good option to provide objective data on this class of players. However, the results obtained in these studies are still scarce, as to quantity or methodology used.

All studies had well-defined goals and targets, as well as methodology. Although, even if the participants’ characteristics have been included, only one justified the sample size. In general, the strategy, the sample size and motivation for the quantity of participants were not related or described in its totality. The results of the analyses were presented and, in its majority, the discussions and conclusions were justified by the results, however, just two [24,28] discussed the limitations of their findings. In addition, just two other studies [27,28] presented to have ethical approval or consent of the participants. It is highlighted that identifying the limitations of the study is important for the design of other questions to be answered.

After the updating and adjustment of the Official Manual of Classification of the IWBF/2021 in relation to the Code of Classification of the IPC/2015, there were considered eligible to play WCB who have impaired muscle strength, impaired range of passive movement,

partial or total absence of lower limb, difference on the length of lower limb, hyposthenia, ataxia or athetosis, presenting conditions that reduce or eliminate their volunteer capability of performing movements [1].

According to the rules of the IWBF [1] players the functional classification is defined in an individual way from the analysis of the motor shred which the players still have to execute movements that are extremely necessary for the modality and that are determiners for the sports performance, as the free-throw shot. Thus, it is necessary to understand the kinematic pattern of free throw by athletes and the influence different impairments on the yield of such fundament. When analyzing the kinematic of the joints involved in the movement of free-throw shots performed by athletes of all functional classes, there were found a few tendencies of movement by Malone et al. (1994) and were supported subsequently by Malone et al. (2002), Goosey-Tolfrey et al. (2002) and Pérez-Tejero et al. (2016). The pattern of pushing the ball was more predominant in low classes (classes 1.0 and 2.5) and another of throwing the ball, predominant in the high classes (classes 3.0 – 4.5). The former is described as a continuous movement, in which the joints move cooperatively with the progression of the technical gesture, including after the ball throw, denoting a more rectilinear trajectory. While the latter should denote proximal-distal sequencing pattern, in which the joints move progressively, producing a more arcuate trajectory.

The sequential movement pattern, predominant in high classes, is described as close to the pattern used by Olympics basketball professional players, who performed a synergic non-simultaneous movement among the shoulder, elbow and wrist joints with the objective to transfer the energy in a more efficient way for the throw [29,30]. The use of this pattern is due to a greater capability of producing strength and balance, for the trunk isometric muscle strength and balance vary among the distinctive class of players, being it greater in high classes [31].

With the characteristics cited above and aiming the successful free-throw shots, the sequential pattern may be a problem to low class players who have greater functional limitations, lesser capability of producing strength and balance [31]. Thus, these players use a pattern of pushing the ball, performing a trunk horizontal inclination on the back, supporting the back on the seat, and performing a slight knot on the head aiming to compensate the balance loss [23,27]. In addition to these strategies, clear distinctions were found in the joint and ball-throw parameters, targeting to perform better throws.

The ball-throw parameters (throw height, throw angle and throw speed) are considered by the literature as kinematic parameters are more determining to a successful throw [32], being then defined by the kinematic and kinetic interrelation of the joints involved in the motor gesture, permeating since the muscle action to throw result [33]. In WCB there is a clear distinction among the classes when observing the ball-throw patterns. Low-class players (classes 1.0 and 2.0) present a lower height in throwing the ball, however, the speed and angle of throw were greater than of those of high class (classes 3.0 and 4.0) [23–25,27].

Ball-throw height is measured by the vertical distance between the ground and the center of the ball [25]. Given this, it is, in an intra-individual way, in WCB, one component that denotes little variation, for it is influenced by the players’ physical characteristics, the trunk length and upper limb, in addition to their equipment [24], for they do not execute the jumping as it happens in Olympic basketball. This because, for a greater stability due to the muscle strength and trunk action volume reduction, the lesser the class, the lesser the chair height.

In addition, the lesser the height of the ball throw during the shot, the greater is the minimum throw angle and the flight trajectory which the ball must cover in the shot, requiring a greater production of throw strength and speed [13,32,34]. That should lead to an advantage on the shot performance for high-class players, who should present not only a greater error margin, but also a greater ball-throw angle, generating a lesser necessity for precision [11].

According to the verified, the ball-throw angle was slightly greater for low-class players [23,25]. However, in a divergent way, there were found in Goosey – Tolfrey et al. (2002) similar angles in low class as well as high class, not being found statistically significant differences and effect size equals zero, which differs from Malone et al. (2002) in which were found statically significant differences (p <  0.01) among high and low classes. Thus, it is necessary that the throw height and subsequently the athlete’s height are described to minimize the variation on the data of these studies.

However, although divergent throw angles, there were found in Malone et al. (2000) greater predominance of short shots by class 1.0 (54% of the total) and a general characteristic in all classes (43% of the total). Nevertheless, in case the necessary throw speeds for a player to reach a minimum throw angle were not achieved and the error margin was exceeded, the shot will not be successful [25]. This supports the previous findings, for, due to the absence of jumping and the consequences of lesser capabilities of strength production, when compared with Olympic basketball athletes, shorter throws will be produced.

Thus, greater ball-throw angles, with a proper strength production, generate greater entrance angles and error margins [32,33,35], performing successful shots. In these studies, the results verified that low-class players presented a higher speed of throw aiming to reach greater throw angles.

In this way, this increase seems to be a strategy that low-class athletes’ use to compensate not only the short ball-throw height, but also the impaired trunk strength, which affects the strength to obtain successful shots. This is because the error margin decreases as the class increases. As the error margin the horizontal distance which the center of the ball may be far from the center of the rim and yet go through the basket [25] higher values of such variable benefit the thrower. While the entrance angle is the angle formed by the tangent to the way of the ball mass center and the horizontal when the ball goes through the rim plan [25], values next to 90° allowed a greater angle for the ball passage, demanding lesser precision and benefiting the thrower [35].

As stated earlier, low-class players need a greater strength production to reach the ideal ball release parameters. Subsequently, they tend to produce greater shoulder and elbow angles during the throw, although it is not what happens, for they do not have the trunk function impaired, high classes present greater throw joint angles in relation to low classes, as to the position of the shoulder as well to the elbow, which justifies the movement pattern when throwing the ball. As low-class athletes denoted greater wrist joint amplitude during the throw, according to Goosey-Tolfrey and collaborators (2002). These data are supported by Limroongreungrat et al. (2010) who found, as they analyzed the maximum angles of the upper extremity of the free-throw shot, a wrist flexion peak by class 3.0 athletes of 19.2° and of 10.2° for class 4.0 athletes.

The position of shoulder, elbow and wrist describes and justifies the characterization of the movement found when pushing and throwing the ball. This because the means found for the shoulder position at throwing was greater for high-class athletes, which indicates that a greater shoulder flexion at throwing, being that Malone and collaborators (2002) presented significant differences between high and low classes.

Yet, according to what was stated previously, in relation to the elbow position at throwing, the means found was that high-class players presented a slightly greater elbow extension. Although no study had found significant statistic differences for this movement, these data align themselves in literature, for all applied protocols were performed on the free-throw line, not altering the throw distance, which minimizes the necessity of executing greater elbow extensions [34].

As to angle speeds, just Malone et al. (2002) verified maximum angle speeds at the throwing, while only Goosey-Tolfrey et al. (2002) obtained mean angle speed at the throw. To Malone and collaborators (2002), significant differences were found for shoulder maximum angle speed among class 2.0 and high-class players and 2.0. Elbow maximum angle speed tends to decrease as the classes increase, being noted significant statistic differences between class 1.0 and high classes, and class 2.0 and class 4.0. As wrist maximum angle speed tend to increase with the class, although not denoting significant statistic difference.

To reach greater ball-throw speeds, as it occurs among low-class athletes, shoulder and elbow maximum angle speed increases, due to the demand of greater strength production, but subsequently it occurs a decrease of wrist maximum angle speed. This may be related to a relative increase on the throw vertical distance among low classes, for being at an inferior height, which should cause an increase on the peak of such variables [11,33,36]. Furthermore, low classes denote a more flexed initial angle of the elbow, the lever arm should be shorter, increasing the elbow amplitude during the movement. Besides that, with a more flexed initial position, the lever arm should be shorter, minimizing the balance loss and increasing the necessary strength production to generate the push during the arm elevation, moment which the lever arm should increase and balance decrease.

Although low-class players use the higher maximum angle speed peak of the shoulder and elbow joints to generate the necessary push during a successful motor gesture, as stated earlier, due to the trunk stability, to Goosey-Tolfrey et al.(2002) and Térez – Perejo (2016) the throw means angle speed by high-class players tend to be greater on the shoulder, while low-class athletes denoted a tendency of using higher angle speed of the wrist during the throw. In addition, a lesser curvature of movement on the joints in series (shoulder, elbow, and wrist), as found among low-class athletes, should increase the wrist movement speed [37], as demonstrated in the cited studies.

According to Okubo & Hubbard (2015), when shoulder and elbow angle speeds are lower, at the expense of the lesser capability of contraction of the heart contraction and neuromuscular feedback which should increase the specific body orientation [38], which aids in the production, power and stability, there is a necessity to increase the wrist angle speed to reach the goal, which supports the previous findings and the movement pattern found among low-class athletes. Besides, the precision of the free throw may be associated to the variations of elbow, shoulder and hips indicating the importance of upper limb coordination and trunk stability to generate a more stable position at the Ball release [28].

By this, low-class players use predominantly distal joints (wrist), for they offer a greater stability and invariability than the proximal joints (elbow and shoulder) for presenting, in the motor gesture of the throw, a shorter lever arm, allowing well-controlled movements in this region. Perhaps, low-class players predominate the use of wrist joints to reduce the movement variability due to the balance loss that the predominant use of distal joints should cause. A greater variability generally indicates a lesser stability, which would not be ideal for the throw [32,39].

This variability of joints at the final movement may be modified by the sequence of motor commands, which should interfere on the precision to reach the target, which is the final hit or miss [37]. By this, to achieve the ideal ball-throw parameters, players use different motor plans, denoting patterns. For instance, to perform a free-throw shot there are an infinite number of trajectories, which can be reached by different combinations of joint angles, due to the player’s distinctive combinations of muscle actions and distinctive joint contraction ability [37]. Such combinations allow the player to be successful in different game situations; however, better throwers are more consistent and present lesser variability among performed throws in similar condition [32].

Furthermore, in a study conducted by Grenha and collaborators (2022), a greater height and throw angle with a lesser release speed should be a good combination for successful shots. However, the requirements necessary for throwing in a wheelchair and standing are different [13], leading this combination to not be found in the parameters of throwing in any WCB functional class, which justifies stated earlier about possible trajectories and joint angle combinations. By this, Malone et al. (2000) and Shigematsu et al. (2021) searched to determine characteristics of free-throw shot, beside the ones cited in other studies, which relate to the players’ precision and performance at this fundament.

Malone et al. (2000), from a visual observation technique reconciled with a schemal diagram developed for this study, verified the ball-into-the-basket movement patterns dividing them into perfect throws (swish), long and shot success and fails. During the assessments, the findings denoted a hit average rate of 53%, being 52% for class 1.0 athletes; 53% for class 2.0 athletes; 49% for class 3.0 athletes; and 54% for class 4.0 athletes, not denoting a correlation between the throw precision and the functional class. The percentages found support Pérez-Terejo e Arbex (2015), but differ from the ones usually found in Olympic basketball, which range from 70.6% to 81.5%, pointing out that the necessary precision for WCB athletes to reach the joint and ball-throw parameters are different when compared to Olympic basketball athletes [13,40].

Therefore, distinctive factors contribute to precision at free-throw shots in WCB, being the main factor, the aspects related to kinematics, to the height of the athlete in the wheelchair, to the capability of production of strength and trunk balance [28,41]. However, to Shigematsu et al. (2021), in addition to these, hips movement variability is also associated with the free-throw precision, suggesting that there is a greater influence of the pelvic-hip lumbar complex in this population, which had not been reported previously in the literature. There were found significant correlations between the elbow angle speed (r =  -0.537, p <  0.05) and hips angle speed (r =  -0.631, p <  0.05). Between the hip angle and the shoulder angle speed (r =  -0.647, p <  0.05) and hips angle speed (r =  -0,763, p <  0.01). In addition to significant elbow and hips angle speed (r =  0.647, p <  0.05) and shoulder and hips angle speed (r =  0.670, p <  0.05).

These data support the fact that players with greater trunk stability and pelvic control presented greater capability of production of strength and Power on the upper extremities [38,42]. This because the muscles involved in the hip-pelvic lumbar complex (erector spinae, rectus abdominal and latissimus dorsie) are the origin of the kinetic chain of throw movement, activating according to the necessity and amplitude of each impairment, providing a better energy transfer to the upper limb (shoulder, elbow, and wrist) with a greater development of strength and precision at the free-throw shot. By this, players who present control of such muscles tend to use them to perform the trunk flexion to initiate the throw on the frontal plan, while those who do not have such control use the actives to maintain the trunk balance before, during and after the throw [40].

Thus, the mechanics used by WCB, as performing the free-throw shot, denote distinctions among the classes, which allow to characterize the kinematic pattern of the throw performed by WCB athletes, mainly when considering balance, strength and the activation of the muscles in the trunk and upper and lower limb which intermediate joint and ball-release patterns to reach the result. Furthermore, it is suggested that besides an intra-individual variability, it is also possible to identify movement tendencies and characteristics in an inter-individual way among different functional class players.

## Limitations and future perspectives

This systemic review has some limitations. Although the search strategy was based on inclusion and non-inclusion criteria, the studies presented heterogeneous data with a certain diversity in the study design, objectives, and methodology, which limited the synthesis analysis, which may have caused inaccuracies and preventing of a meta-analysis. As well as little quantity of studies and data for some variables may have reduced the strength of the presented evidence. Apparently, research in this area denotes a temporal void, in an opposite way to what happened to Olympic basketball which presented an increasing number of researches related to motor behavior and throw performance. By this, several questions still need an answer so there is not only a more reliable theoretical application related to the functional class process, but also to a more accurate practical application in the area of sports science and yield.

Most studies focus on discrete variables defined at specific moments of movement, neglecting the time course, highlighting the need for new research using time series analyses of continuous variables like arm velocity, wheelchair acceleration, and the ball’s full trajectory, achievable through 3D cinematics and inertial sensors. Additionally, advanced biomechanical analyses, such as principal component analysis (PCA), can reveal shooting patterns across functional classes. Investigating contextual factors like distance, fatigue, defenders, and visual cues through simulations, multivariate statistics, computer vision, game indicators, and biochemical data can further enhance understanding of shooting mechanics in wheelchair basketball across different scenarios.

## Conclusion

The articles included in this study characterize the kinematic pattern of the free-throw shot used by athletes of different functional classes in order to obtain successful throws. There are inter-individual differences in the description of the motor gesture of WCB athletes, which allow them to be stratified them into distinctive functional classes, as well as low and high classes, and that different strategies are relevant to increase precision and performance which is directly influenced by each player’s impairment characteristics. Thus, players with a lesser action volume show greater biomechanical adjustment to execute successful throws compared to players with a greater action volume.

## Supporting information

S1 File Total_searchs_revisao_Hugo.xlsx.
(XLSX)
